# Post hoc subgroup analysis of neoadjuvant gemcitabine plus S1 vs gemcitabine plus nab paclitaxel in elderly resectable/borderline resectable pancreatic adenocarcinoma

**DOI:** 10.1038/s41598-025-23185-7

**Published:** 2025-11-12

**Authors:** Daisaku Yamada, Shogo Kobayashi, Hidenori Takahashi, Yoshifumi Iwagami, Hirofumi Akita, Kei Asukai, Junzo Shimizu, Terumasa Yamada, Masahiro Tanemura, Shigekazu Yokoyama, Masanori Tsujie, Tadafumi Asaoka, Yutaka Takeda, Osakuni Morimoto, Akira Tomokuni, Yuichiro Doki, Hidetoshi Eguchi

**Affiliations:** 1 Department of Gastroenterological Surgery, Graduate School of Medicine, Osaka University, Yamadaoka 2-2, Suita, Osaka 565-0871 Japan; 2 Department of Gastroenterological Surgery, Osaka International Cancer Institute, Osaka, Japan; 3 Department of Gastroenterological Surgery, Toyonaka Municipal Hospital, Toyonaka, Japan; 4 Department of Gastroenterological Surgery, Higashiosaka City Medical Center, Higashiosaka, Japan; 5 Department of Gastroenterological Surgery, Rinku General Medical Center, Izumisano, Japan; 6 Department of Gastroenterological Surgery, Hyogo Prefectural Nishinomiya Hospital, Nishinomiya, Japan; 7 Department of Gastroenterological Surgery, Osaka Rosai Hospital, Sakai, Japan; 8 Department of Gastroenterological Surgery, Osaka Police Hospital, Osaka, Japan; 9 Department of Gastroenterological Surgery, Kansai Rosai Hospital, Amagasaki, Japan; 10 Department of Surgery, Japan Community Health Care Organization Osaka Hospital, Osaka, Japan; 11 Department of Gastroenterological Surgery, Osaka General Medical Center, Osaka, Japan

**Keywords:** NAC, R/BR-PDAC, GEM + nPTX, GEM + S-1, R-PDAC, Over 75 years old, Cancer, Cancer therapy, Surgical oncology

## Abstract

This study aimed to evaluate the safety and feasibility of two neoadjuvant chemotherapy (NAC) regimens, gemcitabine plus nab-paclitaxel (GA) and gemcitabine plus S-1 (GS), for elderly patients (aged 75 years and older) with resectable and borderline resectable pancreatic ductal adenocarcinoma (R/BR-PDAC). A post hoc analysis was conducted using data from a randomized controlled trial on NAC for R/BR-PDAC (CSGO-HBP-015). Patients were divided into two groups: those aged 75 years and older (7/46 in GS and 16/48 in GA) and those under 75 years. Short-term outcomes, including resection rates, adverse events (AEs), postoperative complications, and the administration of adjuvant chemotherapy, were compared between age groups for both regimens. The incidence of AEs in patients aged 75 years and older tended to be higher than those of younger patients in both chemotherapy arms, but the differences were not statistically significant. However, the resection rates, postoperative complication rates, and the administration of adjuvant chemotherapy were not affected by age. Both regimens showed comparable safety profiles in elderly and younger cohorts. The GA and GS regimens can be safely administered as NAC for R/BR-PDAC in elderly patients without adversely affecting postoperative outcomes. These findings suggest that both regimens are feasible NAC options even for patients 75 years and older, supporting the need for further randomized controlled trials to validate these outcomes in the elderly population.

*Trial registration*. UMIN Clinical Trials Registry UMIN000021484. This trial began in April 2016, and first registration (First Posted date) is 01/04/2016.

**Supplementary Information:**

The online version contains supplementary material available at 10.1038/s41598-025-23185-7.

## Introduction

Pancreatic cancer is a disease with a poor prognosis, and its incidence and associated mortality are increasing^1,2^. As medical advancements progress, the aging population continues to grow, leading to an increase in the number of elderly patients diagnosed with pancreatic cancer. In Japan, approximately 50–55% of all pancreatic cancer cases occur in individuals aged 75 years and older^1^, and this percentage is expected to increase. The incidence rate (4.7 per 100,000) and mortality rate (4.2 per 100,000) of pancreatic cancer are not significantly different^2^, indicating that the disease is extremely difficult to treat. The only potentially curative treatment for pancreatic cancer is surgical resection. However, the disease is often detected at an unresectable stage, which contributes to its poor prognosis. Furthermore, even in cases where resection is possible, previous studies have shown that surgery alone has a very low likelihood of curing pancreatic ductal adenocarcinoma (PDAC). Multimodal treatment, which combines surgery with chemotherapy, has been proven to be the most effective approach^3,4^. In addition to postoperative adjuvant chemotherapy, recent evidence has demonstrated the efficacy of neoadjuvant chemotherapy (NAC)^5–12^. A phase II/III trial (Prep-02/JSAP-05) comparing neoadjuvant GS therapy (gemcitabine plus S-1 regimen) with upfront surgery for resectable PDAC revealed the superiority of the neoadjuvant GS therapy group in terms of the primary endpoint, overall survival (median survival time, MST: 36.72 months vs. 26.65 months; hazard ratio, HR 0.72; 95% confidence interval, CI 0.55–0.94)^5^. Based on these results, GS therapy is currently considered the most promising NAC regimen for resectable PDAC. However, this trial did not include patients aged 80 years and older, and there is currently no evidence on the efficacy of GS therapy as an NAC in elderly patients. Moreover, there are no reports regarding the safety of NAC for resectable PDAC in elderly patients (e.g., aged 75 years and older), underscoring the need to assess the safety and efficacy of neoadjuvant therapy in this population.

When the optimal regimen for neoadjuvant therapy in patients with resectable PDAC among the elderly is considered, GS therapy, which has proven effective in patients under 80 years of age, is a promising candidate. However, although not based on concrete evidence, the National Comprehensive Cancer Network (NCCN) guidelines suggest other potential regimens, including GnP therapy (gemcitabine plus nab-paclitaxel regimen, called ‘GA’ in this study) and FOLFIRINOX therapy as NAC for resectable PDAC, referencing reports on NAC for borderline resectable PDAC.

In reviewing treatment outcomes in elderly patients, the MPACT trial, a phase III study comparing gemcitabine with GA (GnP) therapy in 861 patients with metastatic PDAC, revealed no significant differences in efficacy or safety between patients over 65 years of age and those under 65 years of age, with no upper age limit for enrollment (the oldest patient was 86 years old)^13^. As a result, GA (GnP) therapy is widely used in routine practice for patients with unresectable PDAC aged 75 years and older. In contrast, FOLFIRINOX therapy has a relatively high incidence of adverse events^14,15^, and the treatment is limited to patients who meet specific criteria, including performance status (PS), age, and bone marrow function. This regimen is not routinely used in patients over 75 years of age due to its toxicity. For this elderly population, GA therapy is considered the standard option among strong chemotherapies. While the efficacy of GA therapy in unresectable pancreatic cancer in elderly patients has been reported, there is little evidence regarding its role as neoadjuvant therapy for resectable pancreatic cancer.

Given these considerations, a comparison between GS therapy and GA (GnP) therapy appears to be the most appropriate approach for determining the optimal neoadjuvant regimen in elderly patients with resectable PDAC. However, no trial has directly compared GS therapy with GA (GnP) therapy as NAC for resectable PDAC in elderly patients.

We conducted a clinical trial (the CSGO-HBP-015 trial) without an upper age limit and reported the results^16,17^. Based on this trial, GA (GnP) therapy is expected to show a greater survival benefit than GS therapy; however, the feasibility of both regimens in elderly patients (aged 75 years or older) has not been addressed.

In this study, we compared the safety of neoadjuvant treatment between patients under 75 years of age and those aged 75 years and older, as well as the feasibility of each neoadjuvant therapy in patients aged 75 years and older.

## Methods

### Study oversight

This study was a post hoc analysis of data from our previous multicenter randomized phase II clinical study, CSGO-HBP-015^16^. This study was approved by the institutional review board of Osaka University Hospital (No. 15443) and by the institutional review board of each institution. This study was conducted in accordance with the Declaration of Helsinki and Good Clinical Practice guidelines.

### Patients

**Fig. 1:**
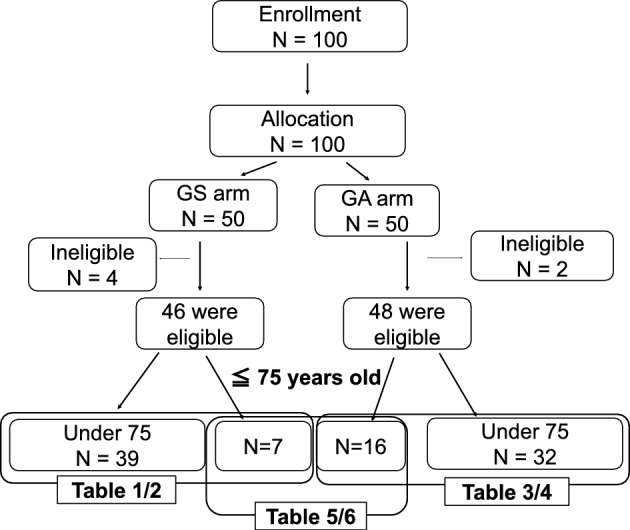
A flow diagram illustrating the results between the treatment arms and comparison cohorts described in each table of this study is presented. *GS arm* gemcitabine + S-1 regimen arm, *GA arm* gemcitabine + nab-paclitaxel arm.

The initial study included 94 eligible patients, 23 of whom were aged 75 years and older. We intended to enroll patients with anatomically resectable PDAC and included patients with resectable PDAC according to our criteria of resectability at the time of the trial. Thus, not only R-PDAC but also BR-PDAC, according to the present classification of the NCCN guidelines (version 2.2021), were included. In the clinical trial evaluating preoperative therapy, 7 patients out of 46 in the GS group and 16 patients out of 48 in the GA group were aged 75 years or older. The flow chart of the participants in this analysis is shown in Fig. 1. First, to compare the safety of each treatment in elderly patients, we compared the short-term outcomes of GS or GA (GnP) therapy between patients under and over 75 years old. Then, we compared the feasibility of these regimens in elderly patients. To refer to elderly patients who underwent surgery without neoadjuvant chemotherapy, we included the other cohort of patients aged 75 years or over who received upfront surgery at Osaka University Hospital.

### Assessment

The resection rate, relative dose intensity (RDI), responses for both NAC arms and adverse events (AEs) were compared. The resection rate was defined as the proportion of resection cases after either NAC treatment. In the reference cohort of patients who underwent upfront surgery, the resection rate was defined as the proportion of patients who underwent radical resection among all patients who initially intended to undergo curative resection. As an evaluation of radiological response, the reduction rate of the tumor diameter in computed tomography (CT) images was evaluated at the time point after NAC performance. The changes in the serum levels of tumor markers were estimated at the same time point as the response to NAC. The pathological response was diagnosed by specialized pathologists at each institution.

### Statistical analysis

For the comparison of the outcomes, the chi-square test and Fisher’s exact test were used. All analyses were conducted with the JMP 14 software program (SAS Institute, Cary, NC, USA). Statistical significance was defined as a two-sided P value < 0.05 (α = 0.05).

**Ethics approval.** This study was a post hoc analysis of data from our previous multicenter randomized phase II clinical study, CSGO-HBP-015^16^. This study was approved by the institutional review board of Osaka University Hospital (No. 15443) and by the institutional review board of each institution. This study was conducted in accordance with the Declaration of Helsinki and Good Clinical Practice guidelines.

**Consent to participate. **Informed consent was obtained from all individual participants included in the study.

## Results

### Comparison of tumor characteristics and treatment response in the GS group

In the GS group, there were no significant differences in tumor characteristics between patients younger than 75 years and those aged 75 years or older (Table 1). Although the incidence of Grade 3 or higher AEs during NAC tended to be higher in the older group than in the younger group, the differences did not reach statistical significance. There were no differences in the RDIs, completion rates, or resection rates. There were no significant differences in terms of tumor size reduction (Supplementary table 1) or CA19-9 reduction rates. Postoperative complication rates and pathological responses also showed no differences between the age groups. Adjuvant chemotherapy was administered similarly across age groups (Table 2).

**Table 1:** Comparison of characteristics between younger (< 75 years) and elderly (≥ 75 years) patients receiving the GS regimen of NAC.

	Under 75 N = 39	Aged 75 and older N = 7	
	*N* or Mean ± SD		*P* value
Age	64 ± 1.2	79 ± 2.8	** < 0.001**
Sex (Male/Female)	19/20	2/5	0.316
Biliary drainage (–/ +)	21/18	4/3	0.872
PS (0/1)	37/2	6/1	0.416
DM (–/ +)	27/12	4/3	0.537
Tumor diameter (mm)	26.1 ± 1.5	22.2 ± 3.5	0.304
Tumor location (Ph/Pb/Pt/other)	20/13/4/2	5/1/1/0	0.187
Ph	20	5	0.324
Pb	13	1	0.313
Pt	4	1	0.752
Other	2	0	0.540
CA19-9 (U/ml)	1337.2 ± 498.9	706.0 ± 1177.7	0.624
CEA (ng/ml)	21.7 ± 14.3	2.6 ± 33.7	0.605
DUPAN-2 (U/ml)	906.5 ± 463.8	2373.6 ± 1037.2	0.204
UICC cT (1/2/3/4)*	11/24/3/1	3/4/0/0	0.612
1	11	3	0.494
2	24	4	0.826
3	3	0	0.448
4	1	0	0.668
UICC cN (0/1 + 2)*	28/11	6/1	0.416
UICC cStage (IA/IB/IIA/IIB/III/IV)*	10/16/2/10/1/0	3/3/0/1/0/0	0.752
IA	10	3	0.352
IB	16	3	0.928
IIA	2	0	0.540
IIB	10	1	0.517
III	1	0	0.668
NCCN R/BR	27/12	7/0	0.088

*GS arm* gemcitabine + S-1 regimen arm, *PS* performance status, *DM* diabetes mellitus, *Ph* pancreas head, *Pb* pancreas body, *Pt* pancreas tail, *NCCN* National Comprehensive Cancer Network, *R* resectable, *BR* borderline resectable.

*TNM classification was performed according to the 8th UICC classification.

**Table 2:** Comparison of clinicopathological findings between younger (< 75 years) and elderly (≥ 75 years) patients receiving the GS regimen of NAC.

Outcomes of NAC treatment			
	Under 75 N = 39	Aged 75 and older N = 7	
	*N*, ratio or Mean ± SD		*P* value
BSA (m^2^)	1.55 ± 0.03	1.48 ± 0.06	0.240
Relative dose intensity of GEM (%)	86.0 ± 3.8	78.5 ± 8.9	0.439
Relative dose intensity of S-1 or nPTX (%)	83.5 ± 4.4	75.5 ± 10.4	0.487
Any grades of adverse events (n, %)**	35, 89.7%	7, 100%	0.239
G3/4 adverse events (n, %)**	29, 74.4%	7, 100%	0.052
Reduction rate of the tumor diameter (%)^§^	− 6.4 ± 3.0	− 20.9 ± 7.2	0.069
Reduction rate of CA19-9 (%)^§^	− 15.5 ± 8.9	− 53.8 ± 21.0	0.101
Reduction rate of CEA (%)^§^	132.0 ± 74.8	28.0 ± 176.5	0.590
Reduction rate of DUPAN-2 (%)^§^	2.8 ± 12.7	− 25.2 ± 29.8	0.393
Completion of NAC (n, %)	23, 59.0%	5, 71.4%	0.527
Resection rate (n, %)	28, 71.8%	5, 71.4%	0.984

Surgical outcomes and adjuvant chemotherapy			
	Under 75 N = 28	Aged 75 and older N = 5	
	*N*, ratio or Mean ± SD		*P* value
PD/DP/TP	18/9/1	2/2/1	0.394
PV/SMV resection	10	0	0.109
Major arterial resection	0	0	-
Operation time, min	482 ± 29.3	395 ± 69.4	0.257
Blood loss, ml	715 ± 191.6	366 ± 453.3	0.484
Surgical morbidity ( +)^§§^	6, 21.4%	2, 40.0%	0.394
POPF ( +)^§§§^	5, 17.9%	1, 20.0%	0.910
Reoperation ( +)	2, 7.1%	0, 0.0%	0.410
Surgical mortality	0, 0.0%	0, 0.0%	–
Adjuvant chemotherapy ( +)	26, 92.9%	4, 80.0%	0.357
Adjuvant chemotherapy (S-1/GEM based)	24/2	3/1	0.283
Completion of adjuvant chemotherapy ( +)	21, 75.0%	3, 60.0%	0.488

Pathological findings in patients with resection			
	Under 75 N = 28	Aged 75 and older N = 5	
	*N* or Mean ± SD		*P* value
R0/R1,2	26/2	4/1	0.405
UICC pT (0/1/2/3/4)*	0/15/12/1/0	0/3/2/0/0	0.804
1	15	3	0.790
2	12	2	0.706
3	1	0	0.668
UICC pN (0/1 + 2)*	10/18	1/4	0.492
Evans classification (I + IIa/IIb + III + IV)	15/13	4/1	0.252
I	3	2	0.093
IIa	12	2	0.905
IIb	11	1	0.335
III	1	0	0.668
IV	1	0	0.668
Number of metastatic lymph nodes	3 ± 0.7	3 ± 1.6	0.768

Severe adverse events observed in each arm			
G3/4/5 adverse events^¶^	Under 75 N = 39	Aged 75 and older N = 7	
	*N* (ratio, %)		*P* value
Hematological	24 (61)	7 (100)	**0.046**
Leukopenia	9 (23)	5 (71)	**0.010**
Neutropenia	21 (54)	4 (57)	0.871
Thrombocytopenia	2 (5)	3 (43)	**0.003**
Anemia	0 (0)	1 (14)	**0.017**
Nonhematological	9 (26)	3 (43)	0.393
Rash	2 (5)	0 (0)	0.540
AST/ALT increase	3 (8)	1 (14)	0.569
Hyperbilirubinemia	0 (0)	0 (0)	–
Febrile neutropenia	1 (3)	0 (0)	0.668
Creatinine increase	0 (0)	0 (0)	–
Anorexia	1 (3)	2 (29)	**0.010**
Constipation	0 (0)	0 (0)	–
Diarrhea	1 (3)	1 (14)	0.161
General fatigue	0 (0)	0 (0)	–
Stomatitis	0 (0)	1 (14)	**0.017**
Hair loss	0 (0)	0 (0)	–
Peripheral neuropathy	0 (0)	0 (0)	–
Others	3 (8)	0 (0)	0.448

*GS arm* gemcitabine + S-1 regimen arm, *NAC* neoadjuvant chemotherapy, *BSA* body surface area, *GEM* gemcitabine, *nPTX* nab-paclitaxel, *NAC* neoadjuvant chemotherapy, *PD* pancreatoduodenectomy, *DP* distal pancreatectomy, *TP* total pancreatectomy, *PV* portal vein, *SMV* superior mesenteric vein, *POPF* postoperative pancreatic fistula, *GEM-based* gemcitabine-based chemotherapy including monotherapy.

**Data on adverse events were collected according to the CTCAE 4.0 classification.

^§^The reduction rates were calculated by dividing the value after NAC treatment by that before the start of NAC treatment. A full RECIST could not be applied because lymph nodes were recorded only for presence/absence of metastases and size changes were not systematically collected.

^§§^The surgical morbidity data were collected according to the Clavien‒Dindo classification, and clinically relevant morbidities (grade IIIa or above) were included in ‘( +)’.

^§§§^The POPF data were collected according to the ISGPF (2016) classification, and clinically relevant POPFs (grade B or above) were included in ‘( +)’.

*TNM classification was performed according to the 8^th^ UICC classification.

^¶^Data on adverse events were collected according to the CTCAE 4.0 classification.

### Comparison of tumor characteristics and treatment response in the GA group

In the GA group, a greater proportion of T3 patients were aged 75 years or older, but there were no significant differences in tumor size between GA patients and younger patients (Table 3). The older group tended to have a higher incidence of Grade 3 or higher AEs during NAC, and the RDI was significantly lower. However, there were no differences in the completion rates of NAC or resection rates between the two age groups. There were no significant differences in terms of tumor size reduction (Supplementary table 1) or CA19-9 reduction rates. No significant differences were found in postoperative complication rates or pathological responses. There were no differences in the administration of adjuvant chemotherapy between the age groups (Table 4).

**Table 3:** Comparison of characteristics between younger (< 75 years) and elderly (≥ 75 years) patients receiving the GA regimen of NAC.

	Under 75 N = 32	Aged 75 and older N = 16	
	*N* or Mean ± SD		*P* value
Age	64 ± 1.1	77 ± 1.5	** < 0.001**
Sex (Male/Female)	18/14	6/10	0.219
Biliary drainage (–/ +)	19/13	11/5	0.524
PS (0/1)	30/2	15/1	1.000
DM (–/ +)	22/10	12/4	0.651
Tumor diameter (mm)	23.0 ± 1.3	23.8 ± 1.9	0.199
Tumor location (Ph/Pb/Pt/other)	22/5/4/1	10/4/2/0	0.717
Ph	22	10	0.665
Pb	5	4	0.433
Pt	4	2	1.00
Other	1	0	0.444
CA19-9 (U/ml)	981.7 ± 445.2	1014.7 ± 629.7	0.966
CEA (ng/ml)	4.1 ± 0.8	4.8 ± 1.2	0.633
DUPAN-2 (U/ml)	370.4 ± 142.5	484.9 ± 223.3	0.668
UICC cT (1/2/3/4)*	11/21/0/0	8/6/2/0	**0.036**
1	11	8	0.297
2	21	6	0.064
3	0	2	**0.041**
UICC cN (0/1 + 2)*	28/4	13/3	0.457
UICC cStage (IA/IB/IIA/IIB/III/IV)*	11/17/0/3/1/0	8/4/1/3/0/0	0.167
IA	11	8	0.297
IB	17	4	0.064
IIA	0	1	0.153
IIB	3	3	0.355
III	1	0	0.475
NCCN R/BR	28/4	12/4	0.273

*GS arm* gemcitabine + S-1 regimen arm, *GA arm* gemcitabine + nab-paclitaxel arm, *PS* performance status, *DM* diabetes mellitus, *Ph* pancreas head, *Pb* pancreas body, *Pt* pancreas tail, *NCCN* National Comprehensive Cancer Network, *R* resectable, *BR* borderline resectable.

*TNM classification was performed according to the 8th UICC classification.

**Table 4:** Comparison of clinicopathological findings between younger (< 75 years) and elderly (≥ 75 years) patients receiving the GA regimen of NAC.

Outcomes of NAC treatment			
	Under 75 N = 32	Aged 75 and older N = 16	
	*N*, ratio or Mean ± SD		*P* value
BSA (m^2^)	1.61 ± 0.03	1.47 ± 0.05	**0.015**
Relative dose intensity of GEM (%)	88.5 ± 3.1	78.1 ± 4.4	0.059
Relative dose intensity of S-1 or nPTX (%)	88.5 ± 3.2	76.4 ± 4.5	**0.033**
Any grades of adverse events (n, %)**	29, 90.6%	16, 100%	0.112
G3/4 adverse events (n, %)**	21, 65.6%	14, 87.5%	0.092
Reduction rate of the tumor diameter (%)^§^	− 18.6 ± 4.8	− 10.5 ± 6.7	0.338
Reduction rate of CA19-9 (%)^§^	− 55.6 ± 9.4	− 40.0 ± 13.1	0.337
Reduction rate of CEA (%)^§^	54.3 ± 35.2	18.5 ± 48.3	0.553
Reduction rate of DUPAN-2 (%)^§^	− 26.1 ± 13.9	9.1 ± 23.6	0.209
Completion of NAC (n, %)	22, 68.8%	9, 56.3%	0.396
Resection rate (n, %)	27, 84.4%	14, 87.5%	0.770

Surgical outcomes and adjuvant chemotherapy			
	Under 75 N = 27	Aged 75 and older N = 14	
	*N*, ratio or Mean ± SD		*P* value
PD/DP/TP	21/5/1	10/4/0	0.524
PV/SMV resection	7	6	0.269
Major arterial resection	0	1	0.160
Operation time, min	487 ± 31.6	484 ± 43.9	0.951
Blood loss, ml	639 ± 99.2	671 ± 392.4	0.852
Surgical morbidity ( +)^§§^	4, 14.8%	4, 28.6%	0.301
POPF ( +)^§§§^	1, 3.7%	3, 21.4%	0.078
Reoperation ( +)	2, 7.4%	1, 7.1%	0.975
Surgical mortality	0, 0.0%	0, 0.0%	–
Adjuvant chemotherapy ( +)	24, 88.9%	11, 78.6%	0.375
Adjuvant chemotherapy (S-1/GEM based)	22/2	9/2	0.395
Completion of adjuvant chemotherapy ( +)	20, 74.1%	9, 64.3%	0.514

Pathological findings in patients with resection			
	Under 75 N = 27	Aged 75 and older N = 14	
	*N* or Mean ± SD		*P* value
R0/R1,2	25/2	13/1	0.975
UICC pT (0/1/2/3/4)*	0/17/10/0/0	1/7/5/1/0	0.074
0	0	1	0.160
1	17	7	0.424
2	10	5	0.934
3	0	1	0.160
UICC pN (0/1 + 2)*	15/12	8/6	0.923
Evans classification (I + IIa/IIb + III + IV)	17/10	11/3	0.299
I	4	7	**0.016**
IIa	13	4	0.228
IIb	10	2	0.129
III	0	0	–
IV	0	1	0.160
Number of metastatic lymph nodes	2 ± 0.5	2 ± 0.7	0.875

Severe adverse events observed in each arm			
G3/4/5 adverse events^¶^	Under 75 N = 32	Aged 75 and older N = 16	
	*N* (ratio, %)		*P* value
Hematological	21 (66)	15 (94)	**0.034**
Leukopenia	14 (44)	8 (50)	0.682
Neutropenia	20 (63)	14 (88)	0.072
Thrombocytopenia	5 (16)	3 (19)	0.784
Anemia	0 (0)	0 (0)	–
Nonhematological	6 (19)	3 (19)	1.000
Rash	1 (3)	0 (0)	0.475
AST/ALT increase	2 (6)	2 (13)	0.460
Hyperbilirubinemia	0 (0)	2 (13)	**0.041**
Febrile neutropenia	0 (0)	1 (6)	0.153
Creatinine increase	0 (0)	0 (0)	–
Anorexia	0 (0)	0 (0)	–
Constipation	0 (0)	1 (6)	0.153
Diarrhea	0 (0)	0 (0)	–
General fatigue	0 (0)	0 (0)	–
Stomatitis	0 (0)	0 (0)	–
Hair loss	0 (0)	0 (0)	–
Peripheral neuropathy	0 (0)	0 (0)	–
Others	3 (9)	1 (6)	0.712

*GA arm* gemcitabine + nab-paclitaxel arm, *NAC* neoadjuvant chemotherapy, *BSA* body surface area, *GEM* gemcitabine, *nPTX* nab-paclitaxel, *NAC* neoadjuvant chemotherapy, PD pancreatoduodenectomy, *DP* distal pancreatectomy, *TP* total pancreatectomy, *PV* portal vein, *SMV* superior mesenteric vein, *POPF* postoperative pancreatic fistula, GEM-based gemcitabine-based chemotherapy including monotherapy.

**Data on adverse events were collected according to the CTCAE 4.0 classification.

^§^The reduction rates were calculated by dividing the value after NAC treatment by that before the start of NAC treatment. A full RECIST could not be applied because lymph nodes were recorded only for presence/absence of metastases and size changes were not systematically collected.

^§§^The surgical morbidity data were collected according to the Clavien‒Dindo classification, and clinically relevant morbidities (grade IIIa or above) were included in ‘(+)’.

^§§§^The POPF data were collected according to the ISGPF (2016) classification, and clinically relevant POPFs (grade B or above) were included in ‘(+)’.

*TNM classification was performed according to the 8^th^ UICC classification.

^¶^Data on adverse events were collected according to the CTCAE 4.0 classification.

### Comparison between the GS and GA groups among patients aged 75 and over

When the GS and GA groups were compared among patients aged 75 years or older, the GA group tended to have a younger age distribution, but no differences in tumor characteristics were observed (Table 5). There were no differences in the RDIs, completion rates, or resection rates. There were no significant differences in terms of tumor size reduction or CA19-9 reduction rates. No significant differences were found in postoperative complication rates or pathological responses. There were no differences in the administration of adjuvant chemotherapy between the two regimens (Table 6).

**Table 5:** Comparison of characteristics between GS and GA in elderly patients (≥ 75 years) and the reference cohort undergoing upfront surgery.

	GS arm N = 7	GA arm N = 16		Ref:Upfront N = 17
	*N* or Mean ± SD		*P* value	
Age	79 ± 0.8	77 ± 0.5	**0.039**	79 ± 1.0
Sex (Male/Female)	2/5	6/10	0.676	11/6
Biliary drainage (-/ +)	4/3	11/5	0.594	11/6
PS (0/1)	6/1	15/1	0.545	–
DM (-/ +)	4/3	12/4	0.399	–
Tumor diameter (mm)	22.2 ± 3.5	23.8 ± 1.9	0.704	21.8 ± 3.0
Tumor location (Ph/Pb/Pt/other)	5/1/1/0	10/4/2/0	0.840	10/5/2/0
Ph	5	10	0.679	10
Pb	1	4	0.567	5
Pt	1	2	0.907	2
CA19-9 (U/ml)	706.0 ± 1177.7	1014.7 ± 629.7	0.742	135.2 ± 35.0
CEA (ng/ml)	2.6 ± 33.7	4.8 ± 1.2	0.357	3.7 ± 0.6
DUPAN-2 (U/ml)	2373.6 ± 1037.2	484.9 ± 223.3	0.240	258.8 ± 109.2
UICC cT (1/2/3/4)*	3/4/0/0	8/6/2/0	0.187	4/5/4/4
1	3	8	0.752	4
2	4	6	0.382	5
3	0	2	0.328	4
4	0	0	–	4
UICC cN (0/1 + 2)*	6/1	13/3	0.792	17/0
UICC cStage (IA/IB/IIA/IIB/III/IV)*	3/3/0/1/0/0	8/4/1/3/0/0	0.725	4/5/7/1/0
IA	3	8	0.752	4
IB	3	4	0.392	5
IIA	0	1	0.499	7
IIB	1	3	0.795	1
NCCN R/BR	7/0	12/4	0.146	16/1

*GS arm* gemcitabine + S-1 regimen arm, *GA arm* gemcitabine + nab-paclitaxel arm, *Ref:Upfront* reference cohort of patients aged 75 and older undergoing upfront surgery, *PS* performance status, *DM* diabetes mellitus, *Ph* pancreas head, *Pb* pancreas body, *Pt* pancreas tail, *NCCN* National Comprehensive Cancer Network, *R* resectable, *BR* borderline resectable.

*TNM classification was performed according to the 8th UICC classification.

**Table 6:** Comparison of clinicopathological findings between the GS and GA arms among elderly patients (≥ 75 years) and the reference cohort undergoing upfront surgery.

Outcomes of NAC treatment				
	GS arm N = 7	GA arm N = 16		Ref:Upfront N = 17
	*N*, ratio or Mean ± SD		*P* value	
BSA (m^2^)	1.48 ± 0.06	1.47 ± 0.05	0.843	1.51 ± 0.03
Relative dose intensity of GEM (%)	78.5 ± 8.9	78.1 ± 4.4	0.971	–
Relative dose intensity of S-1 or nPTX (%)	75.5 ± 10.4	76.4 ± 4.5	0.930	–
Any grades of adverse events (n, %)**	7, 100%	16, 100%	–	–
G3/4 adverse events (n, %)**	7, 100%	14, 87.5%	0.216	–
Reduction rate of the tumor diameter (%)^§^	− 20.9 ± 7.2	− 10.5 ± 6.7	0.321	–
Reduction rate of CA19-9 (%)^§^	− 53.8 ± 21.0	− 40.0 ± 13.1	0.501	–
Reduction rate of CEA (%)^§^	28.0 ± 176.5	18.5 ± 48.3	0.717	–
Reduction rate of DUPAN-2 (%)^§^	− 25.2 ± 29.8	9.1 ± 23.6	0.442	–
Completion of NAC (n, %)	5, 71.4%	9, 56.3%	0.487	–
Resection rate (n, %)	5, 71.4%	14, 87.5%	0.364	14,82.4%

Surgical outcomes and adjuvant chemotherapy				
	GS arm (N = 5)	GA arm (N = 14)		Ref:Upfront (N = 17)
	*N* or Mean ± SD		*P* value	
PD/DP/TP	2/2/1	10/4/0	0.171	10/4/0
PV/SMV resection	0	6	0.077	2
Major arterial resection	0	1	0.539	0
Operation time, min	395 ± 69.4	484 ± 43.9	0.441	457 ± 149.5
Blood loss, ml	366 ± 453.3	671 ± 392.4	0.242	545 ± 160.4
Surgical morbidity ( +)^§§^	2	4	0.637	3
POPF ( +)^§§§^	1	3	0.946	3
Reoperation ( +)	0	1	0.539	0
Surgical mortality	0	0	–	0
Adjuvant chemotherapy ( +)	4	11	0.946	9
Adjuvant chemotherapy (S-1/GEM based)	3/1	9/2	0.770	8/1
Completion of adjuvant chemotherapy ( +)	3	9	0.865	7

Pathological findings in patients with resection				
	GS arm (N = 5)	GA arm (N = 14)		Ref:Upfront (N = 17)
	*N* or Mean ± SD		*P* value	
R0/R1,2	4/1	13/1	0.447	14/0
UICC pT (0/1/2/3/4)*	0/3/2/0/0	1/7/5/1/0	0.407	1/2/11/0/0
0	0	1	0.539	1
1	3	7	0.701	2
2	2	5	0.865	11
3	0	1	0.539	0
UICC pN (0/1 + 2)*	1/4	8/6	0.153	6/8
Evans classification (I + IIa/IIb + III + IV)	4/1	11/3	0.946	-
I	2	7	0.599	
IIa	2	4	0.637	
IIb	1	2	0.764	
III	0	0	–	
IV	0	1	0.539	
Number of metastatic lymph nodes	3 ± 1.6	2 ± 0.7	0.358	3.5 ± 1.0

Severe adverse events observed in each arm				
G3/4/5 adverse events^¶^	GS arm N = 7	GA arm N = 16		
	*N* (ratio, %)		*P* value	
Hematological	7 (100)	15 (94)	0.499	
Leukopenia	5 (71)	8 (50)	0.340	
Neutropenia	4 (57)	14 (88)	0.104	
Thrombocytopenia	3 (43)	3 (19)	0.226	
Anemia	1 (14)	0 (0)	0.122	
Nonhematological	3 (43)	3 (19)	0.226	
Rash	0 (0)	0 (0)	–	
AST/ALT increase	1 (14)	2 (13)	0.907	
Hyperbilirubinemia	0 (0)	2 (13)	0.328	
Febrile neutropenia	0 (0)	1 (6)	0.499	
Creatinine increase	0 (0)	0 (0)	–	
Anorexia	2 (29)	0 (0)	**0.025**	
Constipation	0 (0)	1 (6)	0.499	
Diarrhea	1 (14)	0 (0)	0.122	
General fatigue	0 (0)	0 (0)	–	
Stomatitis	1 (14)	0 (0)	0.122	
Hair loss	0 (0)	0 (0)	–	
Peripheral neuropathy	0 (0)	0 (0)	–	
Others	0 (0)	1 (6)	0.499	

*GS arm* gemcitabine + S-1 regimen arm, *GA arm* gemcitabine + nab-paclitaxel arm, *Ref:Upfront* reference cohort of patients aged 75 and older undergoing upfront surgery, *BSA* body surface area, *GEM* gemcitabine, *nPTX* nab-paclitaxel, *NAC* neoadjuvant chemotherapy, *PD* pancreatoduodenectomy, *DP* distal pancreatectomy, *TP* total pancreatectomy, *PV* portal vein, *SMV* superior mesenteric vein, *POPF* postoperative pancreatic fistula, *GEM-based* gemcitabine-based chemotherapy including monotherapy.

**Data on adverse events were collected according to the CTCAE 4.0 classification.

^§^The reduction rates were calculated by dividing the value after NAC treatment by that before the start of NAC treatment. A full RECIST could not be applied because lymph nodes were recorded only for presence/absence of metastases and size changes were not systematically collected.

^§§^The surgical morbidity data were collected according to the Clavien‒Dindo classification, and clinically relevant morbidities (grade IIIa or above) were included in ‘(+)’.

^§§§^The POPF data were collected according to the ISGPF (2016) classification, and clinically relevant POPFs (grade B or above) were included in ‘(+)’.

* TNM classification was performed according to the 8th UICC classification.

^¶^Data on adverse events were collected according to the CTCAE 4.0 classification.

### Reference to other cohort undergoing upfront surgery

As a reference, we reviewed the other cohort cases from Osaka University Hospital. Among patients aged 75 years or older with R/BR-PDAC who underwent surgery without preoperative therapy during the same period, 17 patients were included (Table 5). At the initial visit to our hospital, the median CA19-9 level was 75, with a resection rate of 82%, and the postoperative complication rate was 21%. Lymph node metastasis was present in 8 patients (57%), with an average of 3.5 metastatic lymph nodes (Table 6).

## Discussion

Both the GS and GA (GnP) regimens were associated with a greater incidence of adverse events in patients aged 75 years and older than in those under 75 years; however, these treatments were administered without compromising resection rates, complication rates, or postoperative outcomes. Additionally, age did not negatively affect the administration of adjuvant chemotherapy even after surgery following NAC treatment. These findings indicate that both regimens are similarly safe as neoadjuvant therapies for patients both under and over 75 years of age.

In the reference upfront surgery cohort, the resection rates were similar, there were no differences in postoperative complications, and no difference was observed in the administration of adjuvant chemotherapy. Based on these short-term outcomes, both GS and GA (GnP) therapies as NAC appear to be safe without significantly compromising perioperative management, even for elderly patients.

In a retrospective study (n = 116) of GA (GnP) therapy for advanced PDAC in patients aged 75 years and older, adverse events occurring within two courses included 8.6% Grade 4 neutropenia, 3.4% Grade 3–4 febrile neutropenia, 2.6% anorexia, and 2.6% rash^18^. These results were comparable to data from nonelderly patients and were consistent with the outcomes observed in our neoadjuvant treatment. Therefore, even among elderly patients with PDAC, neoadjuvant GA (GnP) therapy is considered a tolerable treatment. Administering two courses of GA (GnP) therapy as NAC for resectable PDAC in elderly patients may be a promising approach, potentially improving patient prognosis by suppressing micrometastasis and increasing the rate of R0 resection. Although no trial has directly compared GS and GA (GnP) therapies in patients with unresectable PDAC, a domestic clinical trial reported response rates of 29.3% for GS therapy and 58.8% for GA(GnP) therapy^19,20^. Considering these previous findings, along with the results of our study (CSGO-HBP-015), GA (GnP) therapy may be a viable neoadjuvant option even for patients aged 75 years or older with resectable PDAC. However, this study has several limitations. First, the number of patients aged 75 years or older was relatively small. Second, multiple comparisons were performed in these small subsets without statistical adjustment, so the results should be interpreted with caution due to the risk of false positive findings. Third, although the study primarily included resectable PDAC cases, a small number of borderline resectable cases were also included. Therefore, a randomized controlled trial comparing both regimens as neoadjuvant therapies in elderly patients with resectable PDAC is warranted.

A post hoc analysis of the RCT on NAC for elderly patients with PDAC was conducted. Both the GA (GnP) and GS regimens were considered safe for elderly patients, without having a significant negative impact on postoperative outcomes.

## Supplementary Information

Supplementary Information.

## Data Availability

The data that support the findings of this study are available from the corresponding author (S.K.) upon reasonable request. Individual participant data will not be available. Individual participant data that underlie the results reported in this article, after deidentification, will be shared. Data will be available beginning 9 months and ending 36 months following article publication.
