# Supplementary material for: Post hoc subgroup analysis of neoadjuvant gemcitabine plus S1 vs gemcitabine plus nab paclitaxel in elderly resectable/borderline resectable pancreatic adenocarcinoma
Source: Sci Rep. 2025 Nov 12;15:39597. doi: 10.1038/s41598-025-23185-7 (PMC12612252; doi:10.1038/s41598-025-23185-7)
Supplement: Supplementary file 1 — Supplementary Information. [file 41598_2025_23185_MOESM1_ESM.docx]

**Supplementary table 1** Primary tumor shrinkage evaluated according to RECIST criteria

| Nearly RECIST evaluation* | | | |
| --- | --- | --- | --- |
| Both regimen | **Under 75**  **N=71** | **Aged 75 and older**  **N=23** |  |
|  | *N* (Ratio, %) | | *P* value |
| DCR (CR+PR+SD/PD) | 60/11 (84.5%) | 22/1 (95.7%) | 0.164 |
| CR | 0 (0%) | 1 (4.3%) | 0.077 |
| PR | 21 (29.6%) | 3 (13.0%) | 0.114 |
| SD | 39 (54.9%) | 18 (78.3%) | **0.047** |
| PD | 11 (15.5%) | 1 (4.3%) | 0.164 |
|  |  |  |  |
| GS regimen | **Under 75**  **N=39** | **Aged 75 and older**  **N=7** |  |
|  | *N* (Ratio, %) | | *P* value |
| DCR (CR+PR+SD/PD) | 32/7 (82.1%) | 7/0 (100%) | 0.223 |
| CR | 0 (0%) | 0 (0%) | - |
| PR | 8 (20.5%) | 3 (42.9%) | 0.202 |
| SD | 24 (61.5%) | 4 (57.1%) | 0.826 |
| PD | 7 (17.9%) | 0 (0%) | 0.223 |
|  |  |  |  |
| GA regimen | **Under 75**  **N=32** | **Aged 75 and older**  **N=16** |  |
|  | *N* (Ratio, %) | | *P* value |
| DCR (CR+PR+SD/PD) | 28/4 (78.3%) | 15/1 (93.7%) | 0.504 |
| CR | 0 (0%) | 1 (6.3%) | 0.153 |
| PR | 13 (40.6%) | 0 (0%) | **0.003** |
| SD | 15 (46.9%) | 14 (87.5%) | **0.007** |
| PD | 4 (12.5%) | 1 (6.3%) | 0.504 |
|  |  |  |  |

*Tumor response was evaluated according to RECIST criteria, but only the size change of the primary tumor was assessed. Lymph nodes swelling in several patients were recorded only for the presence or absence, and size changes were not systematically collected in this study. Therefore, the results represent a “nearly RECIST” assessment rather than a complete RECIST evaluation.

DCR, disease control ratio; CR, complete response; PR partial response; SD, stable disease; PD, progression disease; GS regimen, gemcitabine + S-1 regimen arm; GA regimen, gemcitabine + nab-PTX regimen arm
